# Spatio-temporal coordinated development pattern evolution and driving factors of regional population and green economy: Evidence from Shandong Province in China

**DOI:** 10.1371/journal.pone.0304562

**Published:** 2024-07-31

**Authors:** Siliang Guo, Yanhua Diao

**Affiliations:** 1 School of Economics and Management, Qilu Normal University, Jinan, China; 2 School of Economics and Management, Shandong Youth University of Political Science, Jinan, China; Zhongnan University of Economics and Law, CHINA

## Abstract

The study of spatio-temporal evolution characteristics and factors affecting the coordinated development of population and green economy (CD_PGE) in Shandong province, China, has significant decision-making implications for promoting high-quality and sustainable regional development. Based on 2001 to 2020 panel data for each city and economic zone in Shandong province, this paper constructs an evaluation model for the CD_PGE systems. Using growth elasticity models, geographic concentration models, kernel density estimation models, spatial autocorrelation, analysis of population and regional green economy development in Shandong Province from the perspective of spatial agglomeration coupling, spatial and temporal coupling coordination patterns, and evolutionary characteristics. In addition, we use the fixed effect models and panel quantile models to empirically test the effects of coordinated demographic and green economy development. The results show that: (1) In terms of demo-graphic and economic development characteristics, Shandong’s demographic and green economy development trends are good, but there are still many challenges. (2) According to the time series evolution and spatial distribution characteristics, the degree of CD_PGE in Shandong Province is on the rise, and the level of spatial distribution is distinct. (3) From the spatio-temporal dynamical grid evolution of the degree of CD_PGE, the CD_PGE is characterized by significant spatial clustering, but with large regional differences. (4) From an impact factor perspective, both market mechanisms and government intervention have a significant impact on the degree of CD_PGE, but the direction and extent of the impact vary.

## Introduction

The green economy is one of the main goals of regional development, and the population is a fundamental driver of economic development, both of which are central indicators of regional differences. As a central issue in sustainable development, the study of the relationship between population distribution and green economic development has long attracted attention [1–3]. Since the reform and opening-up, as China’s economy has grown rapidly, the widening of the divide and the acceleration of population mobility have led to significant changes in the coordination of population and economic distribution. The mismatch between population and green economy caused by the non-synchronized agglomeration of populations and economies has become an influential reason for the loss of spatial efficiency in regions and the aggravation of development disparities [4]. At present, China is in a critical period of industrialization and urban transformation, and is facing problems such as unbalanced regional development and excessive wealth gap caused by unbalanced population and economic clustering. China has set two strategic goals of coordinated regional development and common prosperity. The coordinated development of the regional population and green economy is one of the core elements of the strategy for regional coordinated development and common prosperity. How each region plays to its strengths to achieve coordinated regional development of population and green economy is a major test of the level of government governance and regional development capacity. For Shandong province, which has a significant regional gap, studying the spatial-temporal relationship between population and green economy can help grasp the distributional status and evolution of factors, serving as a reference for formulating regional development policies scientifically, promoting positive interaction between population and green economy, and achieving coordinated regional development. Therefore, based on measuring the level of coordinated development of population and green economy in Shandong Province, this paper explores the spatio-temporal evolution characteristics and influencing factors of coordinated development of population and economy in Shandong Province, so as to provide a decision-making reference for promoting coordinated development in the region.

The classical economists Adam Smith, David Ricardo and Malthus were among the first to study the relationship between population and green economy, but their ideas differed. Adam Smith believed that population growth promoted economic development, but David Ricardo and Malthus were pessimistic about this relationship [5, 6]. From a historical and realistic point of view, Shandong’s development philosophy still adheres to Adam Smith’s basic idea of positive feedback between population and economy, and it has realized the double prosperity of population and economy. Subsequently, different scholars demonstrated the relationship between population and green economy from their own perspectives, and continuously improved the theory of population and green economy. Among them was Simon, an American academic who wrote The Economics of Population Growth, a classic work on the relationship between population and economics [7]. The research content of related achievements has been continuously enriched from qualitative descriptions of the spatial structure of populations and economies to the disclosure of internal mechanisms [8–12]. Moreover, these achievements provide an essential theoretical basis for the study of balanced population and economic development [13]. According to the theory of regional economy, regional differences in geographical environment, market size, economic level and other aspects will inevitably lead to the existence of comparative advantages among different regions, and the coordinated development of the region should be in line with the comparative advantages and give full play to their vital role in regional economic growth [14, 15]. As a result, various scholars have carried out extensive academic research on the spatial relationship between population and green economy in different regions in conjunction with China’s national conditions. The study scales cover the macro-scale of the whole country and provinces to the medium- and micro-scale of urban units [16–21]. Research methods are also becoming more mature, with Taylor coefficients, geographical concentration coefficients, and regional barycenters being used to quantitatively analyze the relationship between population and green economy [22–24]. The re-search perspective has also gradually expanded from a simple study of the spatial distribution relationship between population and economy in China [25–28] to an in-depth exploration of the coordination relationship between population and green economy through co-ordination models and barycentric analysis models [29–31].

In recent years, the study of population distribution and economic development patterns has attracted the attention of many scholars at home and abroad [32–34], and re-search methods have mainly used spatial metrology to analyze the spatial patterns of regional populations and economies. Yu and Cui [35] studied the coordinated development of population and economy in the Changjitu development and opening-up pilot area in Ji-lin province, noting that the population distribution showed a positive correlation with the economic distribution. Xiao [4] used local and municipal regions of China from 1990 to 2010 as samples and included spatial effects to explore distribution trends, influence factors, and regional differences in China’s population and economy, and argued that China’s economic agglomeration during this period was well ahead of its population concentration areas, inconsistency in population and economic distribution has strong spatial clustering and spatial dependence. Xiang et al. [36] suggested that there is a weak correlation between population and economic clustering in the middle reaches of the Yangtze River Economic Belt, with economic clustering increasing faster than population clustering. Wang et al. [37] calculated the characteristics of population and economic distribution in China and noted that the spatial distribution of population distribution and economic structure in China is not uniform, and the degree of economic clustering is higher than the degree of population clustering, and the coupling between population and economy in the eastern, central, and western regions has gradually intensified. Guan et al. [38] studied the spatial coupling of population and economic development in Chongqing to calculate the population geographic concentration, economic geographic concentration and coupling index. At the same time, they used Geoda and ArcGIS software to establish relevant models and concluded that there was a significant difference between population and economic development in this region, and the geographical concentration showed a feature of "one circle is high, and two wings are low". Lian et al. [39] analyzed the evolution of the spatial patterns of population and economy in Northeast China and argued that economic growth played a negligible role in driving population growth, the distribution of population and economy in each region is not balanced, and the analysis of spatial autocorrelation points out that the distribution of population and economy in Northeast China is more scattered. Zhu et al. [40] analyzed the evolution of the spatial structure of urban clusters in the middle reaches of the Yangtze River between 1990 and 2019, and found that its spatial structure evolved from "three centers" to "one core, two centers and multiple clusters", and finally showed a stable pattern of "two core, multiple centers and multiple clusters". Lu et al. [3] analyzed and studied the population-economic growth elasticity coefficients of 13 cities in Jiangsu province and concluded that there is a strong agreement between population and economic growth elasticity in Jiangsu province.

In conclusion, academic research on the coordinated development of populations and economies has mainly focused on the analysis of pattern evolution and equilibria, and there is a lack of systematic research on the evolution of population and green economic patterns and their drivers in conjunction with the latest census data. Considering the complex relationship between population and high-quality economic development, there are two more points that need to be addressed. First, there is a lack of research on the two-way influence between population and green economic development (GED), and theoretical studies on the coupling and coordination mechanisms between the two need to be strengthened. Second, empirical studies of spatio-temporal pattern evolution and drivers of the degree of coordination development of population and green economy (CD_PGE) are still lacking and need to be complemented. The remediation of the above deficiencies will firstly help to reveal the interplay between population development and GED in a more comprehensive way, and deepen the understanding of the two-way interaction law between population and green economy. Second, it is helpful to systematically understand the spatio-temporal pattern of the CD_PGE, and to understand its influencing factors. In this paper, we argue that there is an interplay and coordination between population and green economy development in Shandong Province, and that its CD_ PGE has a distinct spatio-temporal evolution law, whose development and evolution are mainly influenced by government and market factors. Therefore, in this paper, we estimate and analyze the population and GED levels in Shandong Province from 2001 to 2020 and comprehensively analyze the degree of CD_PGE, its spatio-temporal evolution pattern and driving factors. Considering that subjective empowerment can be interfered by human factors, this paper uses entropy weighting method to measure the level of development of population and green economy in different regions to obtain more objective conclusions. Since the coupled coordination degree model allows for a better evaluation and analysis of the development of coordination between two or more subsystems, the model calculations are simpler and the results more intuitive. In this paper, we adopt the coupled coordination model to explain the interaction between the two subsystems of the population and the green economy, and further measure the coupled coordination of the whole system based on this model. Since the kernel density estimate can directly reflect the specific agglomeration location and the degree of agglomeration of the element under study in geographical space, we adopt the kernel density estimate to measure the spatial agglomeration properties of CD_PGE in Shandong. In theory, there could be spatial correlations between the populations and economies of neighboring cities. Therefore, we use spatial autocorrelation analysis to explore the spatial correlation properties of urban CD_PGE in Shandong. In addition, to reveal the influence factor of CD_PGE in Shandong Province as comprehensively and reliably as possible, we performed empirical tests using panel econometric models. The conclusions in this paper can serve as a reference for developing countries and regions to promote coordinated population and green economic development and improve the high-quality and sustainable development of regional economies and societies.

Shandong, with a total population of more than 100 million people on China’s east-ern coast, has a gross domestic product (GDP) of 8.74 trillion yuan in 2022, accounting for 7.3 percent of China’s total GDP and the third largest in the country. In this paper, we have analyzed the spatial structure and evolution of the regional population and green economy of Shandong province, studied the spatial agglomeration and dispersion trends of the population and green economy at different stages, and explored the spatio-temporal coordination of the population and green economy and its evolution laws and drivers, which not only has important practical implications for Shandong and other Chinese provinces, but also has important reference value for other developing countries in the world to formulate sound regional development policies and promote rational population and green economic concentration, and promote the optimal allocation of resource elements and the reasonable layout of related facilities.

The marginal contribution of this paper is the following. (1) It innovates research perspectives on population and green economics. Independent studies of population development and GED have received more attention from many scholars, but there is no literature on population development and GED from the perspective of regional coordinated development. In this paper, we construct an evaluation index system for the CD_PGE from the perspective of regional coordinated development, and estimate the levels of CD_PGE in Shandong province and 16 cities at different times using a coupled coordination degree model, which is more accurate than the previous studies. (2) Improved research methods. Existing studies have mainly used econometric models to test the one-way relationship and heterogeneity of population development on GED, and to reflect the direction, impact mechanisms, and regional variations in the impact of changes in total urban population, structure, and mobility on green economic development. However, survey methods fail to account for coordinated developmental relationships, spatio-temporal evolution, and drivers. We integrate Various analysis methods. Comprehensive use of kernel density estimation to explore the sequential evolution of the CD_PGE. The spatial distribution and evolution of the CD_PGE are revealed by means of geographical concentration and exploratory spatial analysis. Finally, a panel econometric model is constructed to explore the drivers of the CD_PGE. (3) Expanded the research content. Most existing literature is limited to studies of population and GED in a given region or in all provinces and cities, and lacks detailed surveys of typical provinces, as well as studies of population and green economic coordination within provinces. In this paper, we construct a new measurement system for the CD_PGE to reflect the development of population and green economy and the level of their coordinated development. The study focuses not only on the measurement of CD_PGE, but also on the spatial distribution, historical evolution and driving factors of CD_PGE. The contents of this paper can shed more light on distributional dynamics, evolutionary laws, and factors affecting the CD_PGE.

## Theoretical analysis

### The effect of population on GED

Population size affects GED. Throughout history, population growth has had both positive and negative effects on GED. Population growth can have two effects on GED. On the one hand, certain population growth sets the human foundation for the refinement of the social division of labor and the improvement of technical and intellectual skills, and thus the promotion of GED [41, 42]. On the other hand, an increase in the number of people will lead to a significant reduction in the allocation of resources and rewards, which is not conducive to green economic growth [43]. The economists Malthus and Ricardo have already mentioned this in their classic works. In a market economy, the size of the population determines to some extent the size of the market demand, and the rise of the population favors the formation of economies of scale and promotes technological progress. In the process of urbanization, the greater the number of people, the higher the utilization of infrastructure and the smaller the per capita burden of infrastructure construction, which further stimulates infrastructure renewal and promotes the upgrading of industrial structure and subsequent GED. However, when the population base is relatively large, continued expansion and excessive population size growth will hinder GED. Due to the scarcity of resources, the excessive growth in the consumption needs of the population for basic living directly reduces the level of consumption in education, health care and infrastructure. Inhibiting the improvement of the quality of the population leads to large numbers but low quality, which counteracts its role in promoting GED. At the same time, the surplus population not only creates a waste of human resources, but also brings more destabilizing factors to society. This excessive population burden also limits the development of capital and technology-intensive industries [44].

Population quality and structure affect GED. From a population quality perspective, population quality includes three aspects: physical quality, scientific and cultural quality, and ideological and moral quality. The impact of population quality on GED is mainly reflected in the following three aspects. First, improving the quality of the population can drive up the productivity of the workforce. High-quality talent can promote scientific and technological innovation and GED, and is a core force for economic growth [45]. Second, improving the quality of the population can facilitate the transformation of the industrial structure into a knowledge- and technology-intensive one. High-tech talent is an important workforce support in upgrading industrial structures. The higher the level of human capital structure in a city, the more favorable it is for industrial restructuring and upgrading [46]. Third, improving the quality of the population can enable rapid technological progress. Endogenous growth theory states that the fundamental source of green economic growth lies in the accumulation of knowledge, technological progress, and professional division of labor, and that talent is an important vehicle for its realization [47]. From a demographic structural point of view, the population has an important impact on the green economy due to its dual identity as producer and consumer. As one of the most important demographic factors, population structure determines the rate of economic growth, and a reasonable population structure is a prerequisite for ensuring stable economic growth [48]. During a country’s economic development, population structure, in terms of age, urban and rural areas, education, gender and family size, will have a significant impact on economic growth [49].

Population mobility affects GED. Population movements include migrations and population movements. The impact of population movement on GED has both positive and negative aspects. Positive effects include: On the one hand, the influx of large numbers of people provides the destination with a large supply of sufficient and inexpensive labor, which compensates for the shortage of labor supply in the destination. If there is additional top-level technical talent in the mobile population, this would constitute a strong guarantee for a rapid increase in the productivity level of the destination [50]. At the same time, for resettlement sites with elevated population pressure, population relocation is conducive to reducing local employment pressure, improving the efficiency of local resource utilization, and promoting local economic development [51]. On the other hand, the large number of mobile people in the destination will constitute a huge consumer force, driving consumption demand in the destination, driving the development of related industries and accelerating the pace of urbanization in the destination. In addition to meeting their own consumption needs, the emigrant population can send home to their relatives a considerable amount of cash each year, which is essential to promote economic development in emigrant areas [52]. Negative impacts include: For resettled places with limited resources, a mobile population will cause a wide range of problems such as transportation, housing, energy shortages, ecological and environmental imbalances, and social security [53–55]. The large outflow of young and middle-aged workers will also hinder the development of agricultural production in the relocated areas, aggravate the aging of the local population and adversely affect the sustainable economic development of the relocated areas [56].

Population agglomeration affects GED. The effect of population clustering on green economic growth is not always positive. When the degree of population clustering exceeds the optimal size, it has a certain inhibitory effect on economic growth [57]. Population agglomeration can affect urban green economic growth through factors such as urbanization, industry, and human capital, and the direction and magnitude of the final impact will depend on the degree of these factors. Xie and Zhu [58] found through empirical studies on data from 36 countries that population clustering provides a significant boost to economic growth, but each region or country has an optimal degree of population clustering, and economic growth is maximized when the degree of clustering is maintained at the optimal level. Moreover, population structure effects due to population agglomeration can better promote economic growth [59].

### The effect of GED on the population

GED affects population size. According to Malthus, GED and population growth are negatively correlated [24]. When the level of productivity reaches a significant level, social production will shift from being dependent on the quantity of labor to its quality, and the development of population numbers will be somewhat inhibited. And, as the green economy has grown, so have the costs of raising and educating children. At the same time, women’s social status and rights and interests will be further improved along with GED. Factors such as employment and child-rearing costs have led women’s child-rearing behavior to continue to move in the direction of later child-rearing and fewer births. Continued improvements in the pension security system have changed the previous pension model of raising children for old age and ultimately led to a decline in fertility [60]. As a result, GED affects population size by reducing fertility.

GED affects the quality and structure of the population. The improvement of the quality of the population is subject to a variety of factors, but GED plays the most vital role in its improvement. As the green economy continues to develop, the physical, scientific, and cultural qualities of the population, as well as the mental and moral qualities, will increase correspondingly, thanks to the simultaneous improvement of food, medical care, and education. The level of GED determines the survival and development conditions of the population of a country or region and directly or indirectly affects the population structure [61]. For example, GED affects the gender structure of the population due to preference for sons and other ideas, affects medical conditions, then the age structure, affects industrial development, then the employment structure of the population, affects population mobility and then the urban-rural structure.

GED affects population movement and agglomeration. Population movements are influenced by a myriad of factors, such as nature, society and green economics. However, the scale and direction of population movement ultimately depends on GED. The higher the level of GED, the larger the size of the green economy, which plays a decisive role in the carrying capacity of the population. The narrow size of the green economy has left the area with a weak population carrying capacity, prompting the population to move out. In the last century, it has been found that the more economically developed a place is, the more attractive it is to the population and the easier it is to form clusters of people [62]. The proportion of tertiary industries has a more significant effect on population clustering [63] and the agglomeration of education, medical and other resources and tertiary industry has a more obvious effect on attracting talents [64].

In summary, the relationship between population and green economy is a coordinated development of the coupling of relevant influences. Population development is not only the foundation of GED, but also its ultimate goal. For a certain demographic situation, it is necessary to have a corresponding level of GED to accommodate it, to provide more and better material and spiritual needs for the development of the population, so that promoting the healthy development of the population and the coordinated development of the population and green economy. The population is the foundation and mainstay of GED, and the situation of the population must be continuously adapted to the needs of GED, that is, a certain amount, quality and structure of the population should be provided for GED in order to promote GED. In this way, the green economy and population can develop in harmony. Consequently, population and economic development are interdependent, interacting and interpenetrating. It is only when population and GED are adapted to each other that coordinated development can be promoted. Population should be adapted to GED and vice versa, and the green economy should be adapted to population development. Coordinated development of the two can lead to tremendous development of productivity, and the higher their coordination, the faster and better the optimal allocation of production resources. The two adapt to each other and promote each other, forming a virtuous cycle system for harmonious development.

### Analysis of factors affecting the coordination of coupled population and green economy

New economic geography incorporates spatial factors into the analytical framework of general equilibrium theory and argues that diffusion and externality, backward and forward linkages of industries, and increasing returns to scale can promote industrial agglomeration [14, 65]. Transport costs and workforce mobility are key factors in the spatial clustering and diffusion of economies [66]. The interplay between the centripetal forces generated by increasing return to scale, transportation costs, and production factors is the key factor that causes two regions with the same initial conditions to evolve into a core-periphery industrial agglomeration model [67]. The spatial agglomeration of economies generates externalities that induce the movement of factors such as capital and population. With the inflow of production factors, economic agglomeration will be further strengthened. When capital externalities and labor migration increase through regional integration, large-scale industrial agglomerations will occur spatially [68]. The new economic geography emphasizes the role of agglomeration effects on economic development. The flow of production factors exists in a process similar to the "Matthew effect", and economic agglomeration will further promote the industry to be highly concentrated in the inflow of capital and population [69]. Agglomerative economies give rise to causal and cumulative recurrent mechanisms in regions with identical initial conditions. A small change in conditions will induce a continuous flow of capital and population factors and eventually lead to a high concentration of industries in areas where the factors of production are inflow, leading to an imbalance in the spatial distribution of production and population. Markets and governments are the two dominant forces driving inter-regional resource allocation, and the spatial distribution of populations and economies is in part a result of resource allocation. The evolution of the spatial pattern of population and green economy is mainly driven by the movement of people and capital, which are important production factors that drive economic development. As the dominant force in the allocation of factor resources, the market and the government play different roles in the allocation of factors, with distinct differences in guiding the flow of population and capital production factors. The market mechanism emphasizes the role of competition and production efficiency, and promotes the concentration of production factors to developed regions with high returns on capital. When the market mechanism plays its full role, it will bring problems such as regional development differences and loss of social benefits. Therefore, the government will exercise its macro-control role and adopt relevant policies to actively intervene in the market to adjust the differences between regional developments. The role of market mechanisms and governments will have an important impact on the spatial distribution of people and economies. Therefore, based on the agglomeration economic theory of new economic geography and existing relevant literature, this study analyzes the main factors affecting the evolution and spatial distribution coordination of population and green economy in Shandong province from the perspectives of the government and the market, which are two major entities affecting the allocation of resource factors.

Market mechanisms emphasize the importance of efficiency in guiding the flow and clustering of production factors. First, market mechanisms facilitate the flow of physical capital and human capital through price mechanisms and competition mechanisms. Due to a series of interdependent causal relationships in economic activities, there will be a wealth "Matthew effect" in which the strong become stronger and the weak become weaker [4]. Under the influence of market mechanisms, it is inevitable that the green economy will rapidly become concentrated in areas with good historical foundations. As a production factor, the human capital will also move to places where there is an economic agglomeration, provided there are no barriers to the movement of the human capital and the market mechanism plays its full role. However, most population movements are induced by economic factors. Compared to economic agglomeration, population agglomeration has a time lag to some extent, so there is some deviation between population and economic agglomeration. Therefore, in this paper we assume that the role of market mechanisms will further exacerbate the inconsistencies in population and green economic distribution. Green economic clustering will inevitably occur in the development of regional economies, but excessive economic clustering and lagging population clustering will exacerbate the imbalance between regions, which is not conducive to the CD_PGE.

In terms of government management, reasonable government intervention is an important means for transition economies to achieve economic growth and promote CD_PGE. The government can formulate appropriate regional population and green economic development policies and continuously optimize the regional institutional environment. Meanwhile, the government will allocate resources such as science, technology and education at different stages of regional development according to different regional development strategies to promote CD_PGE [70]. In addition, transportation infrastructure is an important policy variable affecting CD_PGE.

## Research design

### Study area introduction

Shandong is in China’s eastern coastal provinces. The land area is 114°48′~ 122°42′ east longitude, 34°23′~ 38°17′ north latitude, east-west length of 721.03 km, north-south length of 437.28 km. The territory consists of a peninsula and two inland parts. The Shandong Peninsula protrudes from the Bohai Sea and the Yellow Sea, and confronts the Liaodong Peninsula at a distance. The inland part borders Hebei, Henan, Anhui and Jiangsu provinces from north to south. With an area of 155,800 square kilometers on land and 159,600 square kilometers at sea, Shandong will have jurisdiction over 16 cities and 136 county-level units in Jinan, Qingdao, Zibo, Zaozhuang, Dongying, Yantai, Weifang, Jining, Tai’an, Weihai, Rizhao, Linyi, Dezhou, Liaocheng, Binzhou and Heze by the end of December 2022.

### Indicator measurement

#### Population development indicators and GED indicators

Table 1 shows the system of indicators for population development and GED. Following the principles of availability, scientificity, representativeness and systematization, this paper examines the relevant academic research results of former students [71–73], and combines the actual development situation of Shandong province to build a population—green economic evaluation index system of Shandong province, which consists of 1 target layer, 2system layers and 16 indicator layers.

**Table 1:** Population and green economic integrated evaluation index system.

Target layer	System layer	Indicator layer	Unit
		Natural growth rate	%
		population gross	Ten thousand people
		Population density	people per square kilometer
		Urban and rural population ratio	%
	Population system	Gender ratio	%
		Population urbanization rate	‰
		Number of employees	Ten thousand people
Population—Green economic evaluation index system		Employment in urban units	Ten thousand people
		GDP	100 million yuan
		The proportion of secondary industry in GDP	%
		The proportion of tertiary industry in GDP	%
	Green economic system	Per capital GDP	Yuan
		Per capita total retail sales of consumer goods	Yuan/person
		Energy consumption per unit GDP	Yuan/ standard coal per ton
		Expenditure on environmental protection as a proportion of GDP	%
		Average wages of workers	Yuan

Population systems generally include indicators such as "population level, age structure, gender structure, net population migration, urban and rural structure, medical infrastructure, and educational attainment". To reflect the population situation objectively and comprehensively in Shandong, eight indicators were selected to constitute the population system, which were screened using frequency statistics and expert consultation methods. The green economic system generally includes "green economic aggregate, green economic level, green economic factors, green economic structure" and other indicators. Different green economic indicators reflect different green economic phenomena. To form an organic whole with representative green economic indicators, eight green economic indicators were selected to form a green economic system based on existing research results and the green economic development situation of Shandong province over the past 20 years.

In this paper, we use the entropy weighting method to synthesize the indices of the two systems and calculate the population development index and GED index, respectively.

#### The coupling coordination index of population and green economy

Based on the concept of capacity coupling in physics, the coupling degree model for regional populations and economies is constructed by referring to the capacity coupling coefficient model. The formula is as follows.

$$Ci={\left\{\frac{\text{Gi}\times \text{Ei}}{{\left(\left(\text{Gi}+\text{Ei}\right)/2\right)}^{2}}\right\}}^{k}$$ (1)

Where, Ci is the coupling degree, the value range is [0,1]. Gi and Ei denote the population development index and GED index of region i, respectively; Here k is the adjustment coefficient and, in this paper, k = 2.

The coupling degree cannot be used to judge the absolute level of the two systems, so in this paper we use a coupling coordination degree model based on the coupling degree, formulated as follows.


$$DI=\sqrt{Ci\times Ti}\;\;\;Ti=\alpha Gi+\beta Ei$$ (2)

In the formula, DI is the coupling coordination degree and its value range is [0,1]. The larger the value, the better the coordination between the two systems. Ti is a composite coordination index; Since both are equally important, α = β = 0.5.

### Influential factor variable selection

#### Explained variable (DI)

In this paper, the coupled coordination degree index of population and green economy (DI) is used as a proxy variable to reflect the level of CD_PGE in Shandong province.

#### Explanatory variables

1. Capital level. Based on the above theoretical analysis, the unbalanced flow and concentration of material and human capital is the main factor causing the gap between regional population and green economy development. So, this paper considers the level of physical capital (CAPI) and human capital (HUMAN) as variables that reflect the core elements of the market. Referring to the related literature [74], in this paper, we use per capita fixed asset investment as a surrogate variable for physical capital status and reflect the level of human capital in a region as a proportion of the number of ordinary college students in the resident population.
2. The level of government intervention. Governmental mechanisms promote the spatial evolution of population and green economic agglomerations through their effect on the allocation of factor resources. On the one hand, government intervention is an important means to promote CD_PGE. On the other hand, transport infrastructure is an important support for the coordinated development pattern of the regional population and green economy. Therefore, in this study, the intensity of government intervention (GOV) and the status of transport infra-structure (TRAF) were selected as two core variables that reflect the impact of government intervention on the coordinated development of population and green economy.

The government affects the regional population and green economy through different policy means and then changes the spatial distribution pattern of population and green economy in the region, while government intervention plays a role through changes in fiscal revenues and expenditures. Therefore, the proportion of fiscal spending in regional GDP is used as a proxy variable in this study to reflect the intensity of government intervention.

Improvements in transportation facilities are conducive to reducing the cost of movement of people and other factors, and thus to the movement and clustering of people, while improvements in transportation facilities can reduce the cost of transportation transactions and facilitate green economic clustering. If the improvement of transportation facilities has a stronger effect on population clustering than green economic clustering, it will promote the level of coordinated development of population and green economy, inhibit the coordinated development of population and green economy [75]. As a public good provided by the government, transport infrastructure can replace the level of government intervention to a certain extent. In this study, the density of roads in a municipality is adopted as a proxy variable to reflect the level of regional transportation infrastructure construction, and the total number of highway miles per 100 km^2^ is used specifically for this calculation.

#### Control variables

The CD_PGE is a comprehensive process that will be influenced by many complex factors. Since this study mainly analyzes factors affecting the CD_PGE in Shandong province from the perspective of market mechanisms and government mechanisms affecting the flow of factors, it is necessary to include some important control variables in the model. With reference to the relevant literature [76–78], the green economic development level (PGDP) and industrial structure (IS1 and IS2) are chosen as control variables in this study. The per capita GDP of the region reflects the level of GED, and the ratio of the value of output of secondary industries in GDP (IS1) to the value of output of tertiary industries in GDP (IS2) reflects the industrial structure of the region.

### Model and method

#### Population-green economic growth elasticity

Population-green economic growth elasticity is the ratio of unit population change to unit total green economic change over a period and is used to reflect the degree to which population growth and green economic growth are coordinated. The precise formula for the calculation is as follows.

$$E_{i}=\frac{\Delta p_{i}/p_{i}}{\Delta G_{i}/G_{i}}$$ (3)

Where, *E*_*i*_ represents the elasticity coefficient of population-green economic growth of region i, Δ*p*_*i*_ represents the month-on-month change of the permanent population of region i, *p*_*i*_ denotes the total permanent population of region i, Δ*G*_*i*_ represents the month-on-month green economic change of region i, and *G*_*i*_ is the GDP of region i.

#### Geographical concentration

Geospatial concentration is used to describe the distribution of geospatial elements in a certain region at a certain point in time. In this paper, we use the degree of geographical concentration of population and green economy to characterize the dynamic spatial distribution and agglomeration of population and green economy in various cities of Shandong province over a certain period. The formula are as follows.


$$R_{pi}=\frac{p_{i}*\sum p_{i}}{a_{i}*\sum a_{i}}$$ (4)

$$R_{Gi}=\frac{G_{i}*\sum G_{i}}{a_{i}*\sum a_{i}}$$ (5)

However, population and green economic geographic concentration can only represent spatial agglomeration relations of a single factor. To better show the contrast between population and green economic agglomerations, the relative coupling index of population and green economy is introduced here to reflect the relative relationship between population and green economy in spatial agglomerations and thus to obtain the relative development status of the two. This value is formally expressed as the ratio between population and green economic geographic concentration, reflecting the comparative relationship between population and green economic development in spatial agglomerations of cities. The formula is as follows.


$$Z_{i}=\frac{R_{pi}}{R_{Gi}}$$ (6)

Here, *R*_*pi*_ and *R*_*Gi*_ denote the geographic concentration of population and green economic concentration in region i, respectively. *Z*_*i*_ denotes the relative coupling index of population and green economy in region i. *p*_*i*_, *G*_*i*_, and *a*_*i*_ denote the population, gross regional product, and land area of region i, respectively. The larger *R*_*pi*_ and *R*_*gi*_, respectively, the higher the population density and the higher the level of green economic development in the region. If *Z*_*i*_ is less than 1, it means that the green economic agglomeration lags behind the population agglomeration. If *Z*_*i*_ is greater than 1, it means that the green economic cluster is ahead of the population cluster. The more *Z*_*i*_ approaches 1, the higher the CD_PGE agglomerations.

#### Kernel density function model

The kernel density function is a nonparametric estimation method with the advantages of being robust and having a weak dependence on the data distribution. Moreover, it has been widely used in the study of spatially distributed agglomerative states, and this value is positively correlated with the degree of agglomeration of spatial variables. To better study the spatial distribution properties of CD_PGE in Shandong Province, we analyze it using kernel density estimation. In addition, kernel density estimation curves are used to describe the distribution of random variables with continuous density curves to reflect the distributional location, shape, and tractability properties of CD_PGE. The specific formula of kernel density estimation method is as follows [79].


$$f_{N}\left(X\right)=\frac{1}{Nh_{N}}\sum_{i=1}^{N}K\left(\frac{X_{i}-\bar{X}}{h_{N}}\right)$$ (7)

$$\text{K}\left(\text{x}\right)=\frac{e^{-0.5x^{2}}}{\sqrt{2\pi }}$$ (8)

Here *f*_*N*_ (*X*) is the kernel density estimation function for the random variable X. N is the number of cities. *K*(*x*) denotes the kernel function of the variable x. *h*_*N*_ represents the bandwidth, which is a positive number greater than 0 that reflects the estimation accuracy and is proportional to the smoothness of the kernel density curve.

#### Spatial Moran’s index (Moran’s I)

To effectively reflect the spatial agglomeration pattern and characteristics of CD_PGE in Shandong Province, the coupling was analyzed using the global and local spatial Moran’s I indices, respectively. The calculated fomentation is as follows [79].


$$\text{GMI}=\frac{\sum_{i=1}^{N}\sum_{j=1}^{N}w_{ij}\left(X_{i}-\bar{X}\right)\left(X_{j}-\bar{X}\right)}{\sum_{i=1}^{N}\sum_{j=1}^{N}\frac{w_{ij}\sum_{i=1}^{N}{\left(X_{i}-\bar{X}\right)}^{2}}{N}}$$ (9)

$$\text{LMI}=\frac{\sum_{i=1,j\neq 1}^{N}w_{ij}\left(X_{i}-\bar{X}\right)\left(X_{j}-\bar{X}\right)}{\sum_{i=1}^{N}{\left(X_{i}-\bar{X}\right)}^{2}}$$ (10)

Here, *X*_*i*_ and *X*_*j*_ denote the coupling degree between the population and the GED of cities i and j in a given year, respectively. N is the number of cities. *w*_*ij*_ denotes the spatial weight matrix, which is computed in the form of a distance-space matrix.

Moran’s I is generally between [–1, 1]. Under the given significance condition, if the value of Moran’s I is greater than 0, it indicates that the regions under study are spatially positively correlated, that is, regions with the same properties are significantly clustered in the spatial distribution. The closer the value of Moran’s I is to unity, the smaller the difference between the properties of the regions in the spatial distribution. In turn, if the value of Moran’s I is less than 0, it indicates that the spatial correlation of the studied regions is negative, that is, regions with the same properties differ significantly in their spatial distribution. When the value of Moran’s I is 0, the properties between the regions are independent of each other and the regions present a randomly distributed spatial state.

The Moran scatter plot depicts the correlation between the variable Z and its spatial lag vector WZ. The horizontal axis of the scatter plot is the observed value of the variable Z, and the vertical axis is the value of the spatial lag vector, forming four quadrants. The first quadrant indicates that the observed region and the surrounding region have relatively high values of this property, HH type. The second quadrant indicates that the observed region has a lower value of this property and the surrounding region has a higher value of this property, LH type. The third quadrant indicates that the observed region and the surrounding region have relatively low values of the property, LL type. The fourth quadrant represents the high attribute value of the observed region and the low attribute value of the surrounding region, HL type.

#### Panel measurement model

Based on the above theoretical analysis, and in conjunction with the actual development of Shandong province, in this paper, starting from the two main bodies of resource allocation, we use relevant data from 16 cities in Shandong province from 2001 to 2020 as the basis for an empirical analysis of factors affecting the CD_PGE. We constructed a fixed-effect panel data model for factors affecting the CD_PGE in Shandong province.


$$\begin{array}{l} DI_{it}=\beta_{0}+\beta_{1}CAPI_{it}+\beta_{2}HUMANP_{it}+\beta_{3}GOV_{it}+\\ \beta_{4}TRAF_{it}+C_{i}\sum Control_{it}+City+Year+\mu_{it} \end{array}$$ (11)

Here i and t denote city and year, respectively. DI is the coupling index of population and green economy. *β*_0_ is the constant term, and *β*_*i*_ is the regression coefficient of the main influencing factors. *C*_*it*_ is the regression coefficient for each control variable. The City and Year indicate the fixed city and year, respectively. *μ*_*it*_ is a random error term. When the coefficient of the independent variable is positive, indicating that it has a positive effect on the CD_PGE, changes in the explanatory variable will promote the CD_PGE. Otherwise, it is not conducive to the CD_PGE.

The biggest advantage of the fixed-effect model is that it allows for better control of inter-city heterogeneity, which is difficult to observe but does not change over time, and effectively reduces the bias in coefficient estimation due to missing variables. However, it should be noted that the fixed effect model has a wasteful variational degree of freedom that may give biased estimates and is significantly flawed in underestimating the standard error.

Quantile panel data models can combine the advantages of fixed effect models and quantile regression to study the relationship between variables and better analyze the relationship between the conditional distributions of explanatory variables and the explanatory variables at different points in time based on controlling for individual differences. Therefore, to ensure the robustness of the study, we constructed a panel-wise quantile regression model.

$$\begin{array}{l} Q_{\tau }(DI_{it})=\gamma_{0}+\gamma_{1\tau }CAPI_{it}+\gamma_{2\tau }HUMANP_{it}+\gamma_{3\tau }GOV_{it}+\\ \gamma_{4\tau }TRAF_{it}+C_{i\tau }\sum Control_{it}+City+Year+\mu_{it} \end{array}$$ (12)

Where, *Q*_*τ*_ (*DI*_*it*_) is the τ quantile of the population green economic coupling index, and *γ*_*iτ*_ is the tau quantile regression coefficient of the main influencing factors. *C*_*iτ*_ denotes the quantile regression coefficient of τ for the control variables.

### Data source and processing

The population and green economic data used in this paper are derived from the Shandong Provincial Statistical Yearbook 2000–2021, Shandong Provincial Population Census No. 5, Shandong Provincial Population Census No. 6, and Shandong Provincial Population Census No. 7. Data for the other indicator variables were obtained from the local municipal statistical bulletins and the China Urban Statistical Yearbook 2001–2021. Related data for the vector map of Shandong province came from the National Geographic Information Resource Catalog Service. Some missing data was supplemented by linear interpolation to reduce the GDP-related data at constant 2000 prices. The descriptive statistical results for each relevant variable are presented in Table 2.

**Table 2:** Descriptive statistics of variables.

Variables	observations	mean	sd	min	p50	max
**DI**	320	1.002	0.313	0.57	0.922	1.822
**CAPI**	320	10.507	0.818	7.881	10.633	12.165
**HUMAN**	319	11.033	1.017	8.375	10.939	13.497
**GOV**	320	0.095	0.032	0.041	0.09	0.201
**ROAD**	320	0.307	0.213	0.021	0.244	1.231
**IS1**	320	51.655	8.761	27.86	51.255	82.28
**IS2**	320	37.955	9.145	12.87	36.645	61.79

## Analysis of the coordination relationship between population and GED

### Analysis of temporal evolution characteristics of CD_PGE in Shandong province

#### Population and GED aggregate continues to rise

**Fig 1:**
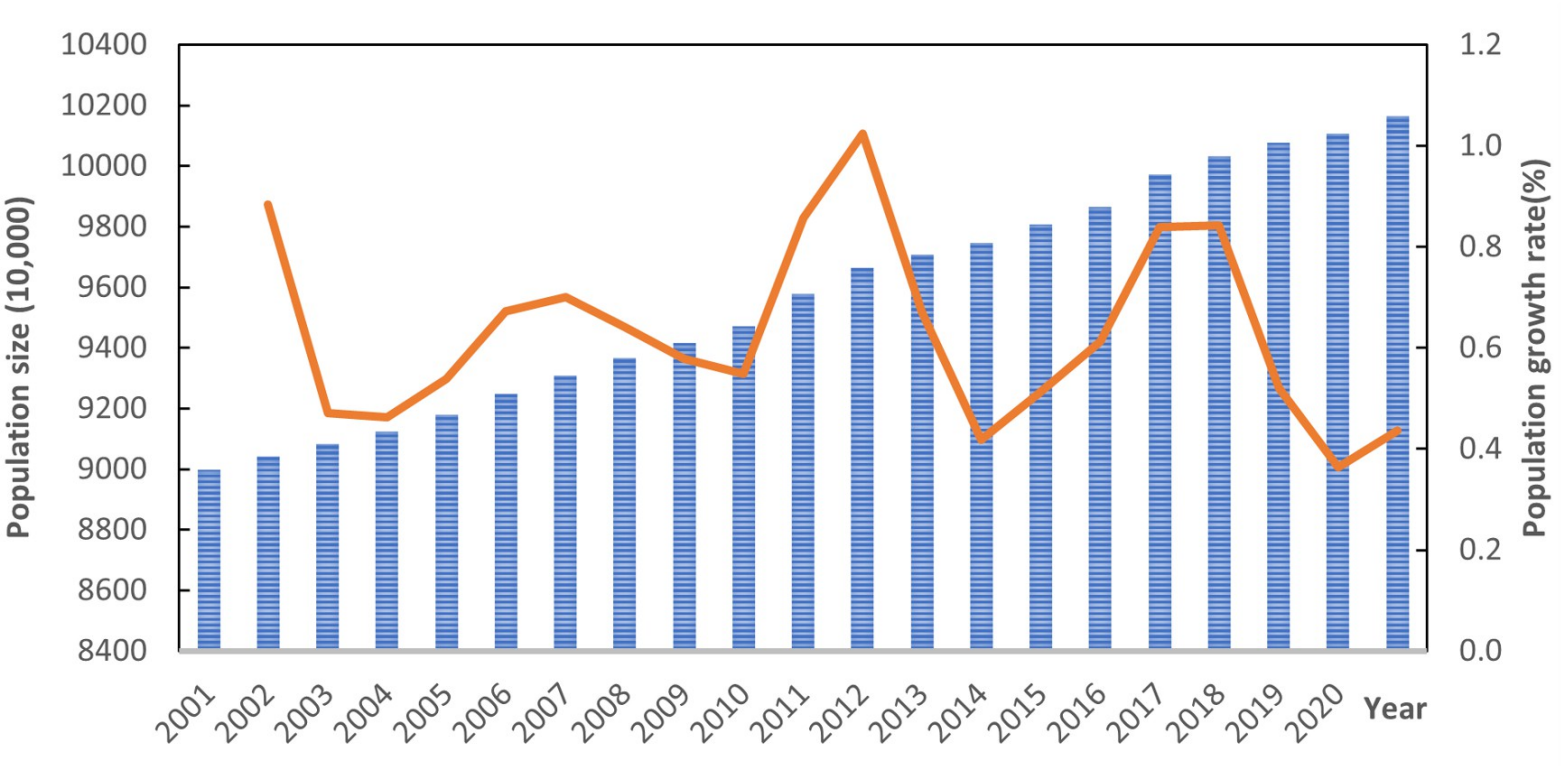
Total population and growth rate in Shandong Province from 2011 to 2020.

Fig 1 shows the total population and growth rate of Shandong province from 2011 to 2020. Over the past two decades, Shandong’s population has expanded steadily, growing by 11.24 million people. The population growth structure is mainly mechanical, and green economic and social development have created a great attraction for people to move to other provinces and regions. In terms of population growth, the province has seen a fluctuating downward trend over the past two decades, with an average annual growth rate of 0.63 percent. The highest peak growth rates occurred in 2001, 2011 and 2017 at 0.884, 1.024 and 0.842 respectively. The 2011 and 2017 spikes were largely due to the fact that large numbers of people not previously counted were added to the total population in the 2000 and 2010 censuses. Population growth rates began to rise after 2015, largely due to the country’s two-child policy.

**Fig 2:**
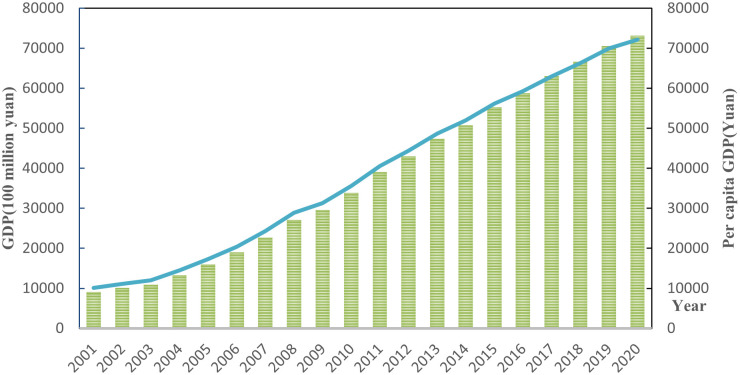
Green economic aggregate of Shandong province from 2011 to 2020.

Fig 2 shows the change in the aggregate green economy of Shandong province from 2011 to 2020. Over the past two decades, green economic indicators in Shandong have increased significantly. Shandong’s GDP grew from 907.622 billion yuan in 2001 to 712.9 billion yuan in 2020, with the aggregate green economy expanding about 8.1 times in 20 years. GDP per capita increased from 9,259.57 yuan in 2001 to 72,151.35 yuan in 2020, an increase of 7.9 times in 20 years.

#### Population and GED elasticity and volatility growth

**Fig 3:**
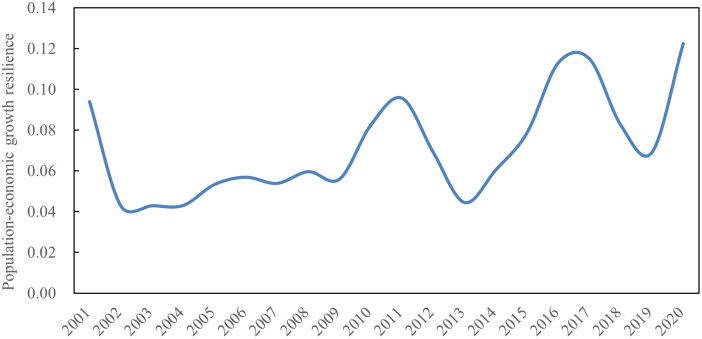
The elasticity of population-GED in the province from 2011 to 2020.

The variation of the elasticity of population-GED in the province from 2011 to 2020 is shown in Fig 3. It can be seen that the elasticity of population and GED in Shandong province has shown an evolutionary trend of fluctuating growth over the past two decades, with a wide range of fluctuations, indicating that the overall level of CD_PGE is not high, but the driving influence of GED on population growth has gradually increased. The average annual elastic coefficient of population and GED in Shandong province over the past two decades is 0.07, meaning that 1 percent GED will drive 0.07 percent population growth.

**Fig 4:**
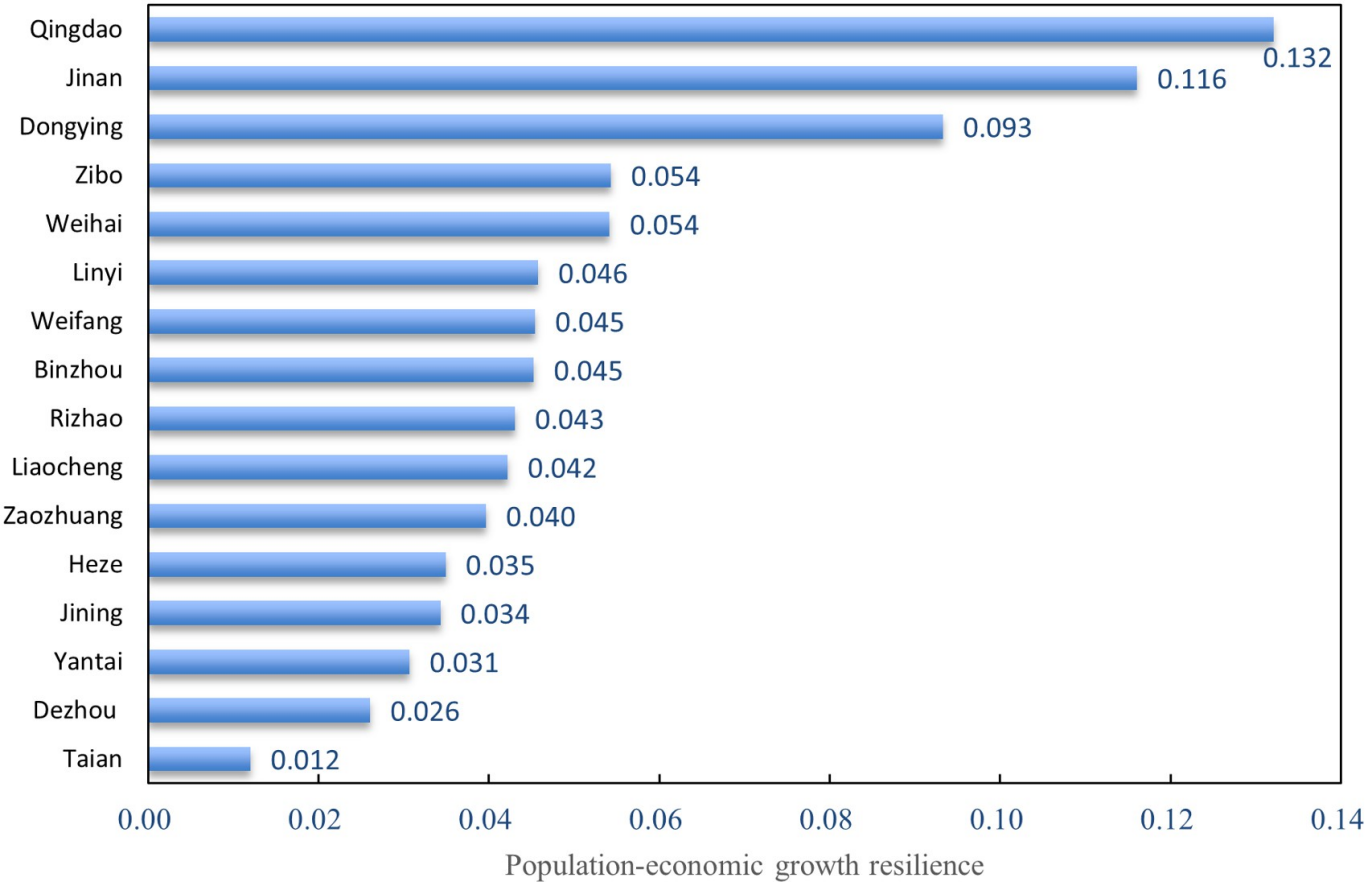
Average annual population-GED elasticity for 16 cities in Shandong province during 2011–2020.

The elasticity of population-GED in 16 prefecture-level cities in Shandong province during 2011–2020 is shown in Fig 4. Overall, there is wide variation in the elasticity of population-GED among the 16 cities, with Tai’an City having the smallest average annual elasticity coefficient of 0.012. The average annual elastic coefficient is largest in Qingdao at 0.132. Specifically, Qingdao, Jinan and Dongying showed significantly better GED than other cities in terms of driving population growth. Qingdao, Jinan and Dongying have elasticity coefficients of 0.132, 0.116 and 0.093, respectively, for population and GED during 2011–2020. In other words, 1 percent green economic growth in Qingdao, Jinan and Dongying will drive local population growth of 0.132 percent, 0.116 percent and 0.093 percent, respectively. As the two poles of Shandong’s GED, Jinan and Qingdao are able to attract foreign populations due to their superior green economic environment and diversified industrial structure. In addition, factors such as well-developed transportation infrastructure and important transportation hubs are also major reasons for the CD_PGE. In Zibo, Weihai, Linyi, Weifang, Binzhou, Rizhao, Liaocheng, Zaozhuang, Heze, Jining and Yantai, the elastic coefficient of the average annual population GED is between 0.031 and 0.06. GED in the 11 cities had a certain driving effect on population growth. GED in the two remaining cities has a more limited role in driving the population.

**Fig 5:**
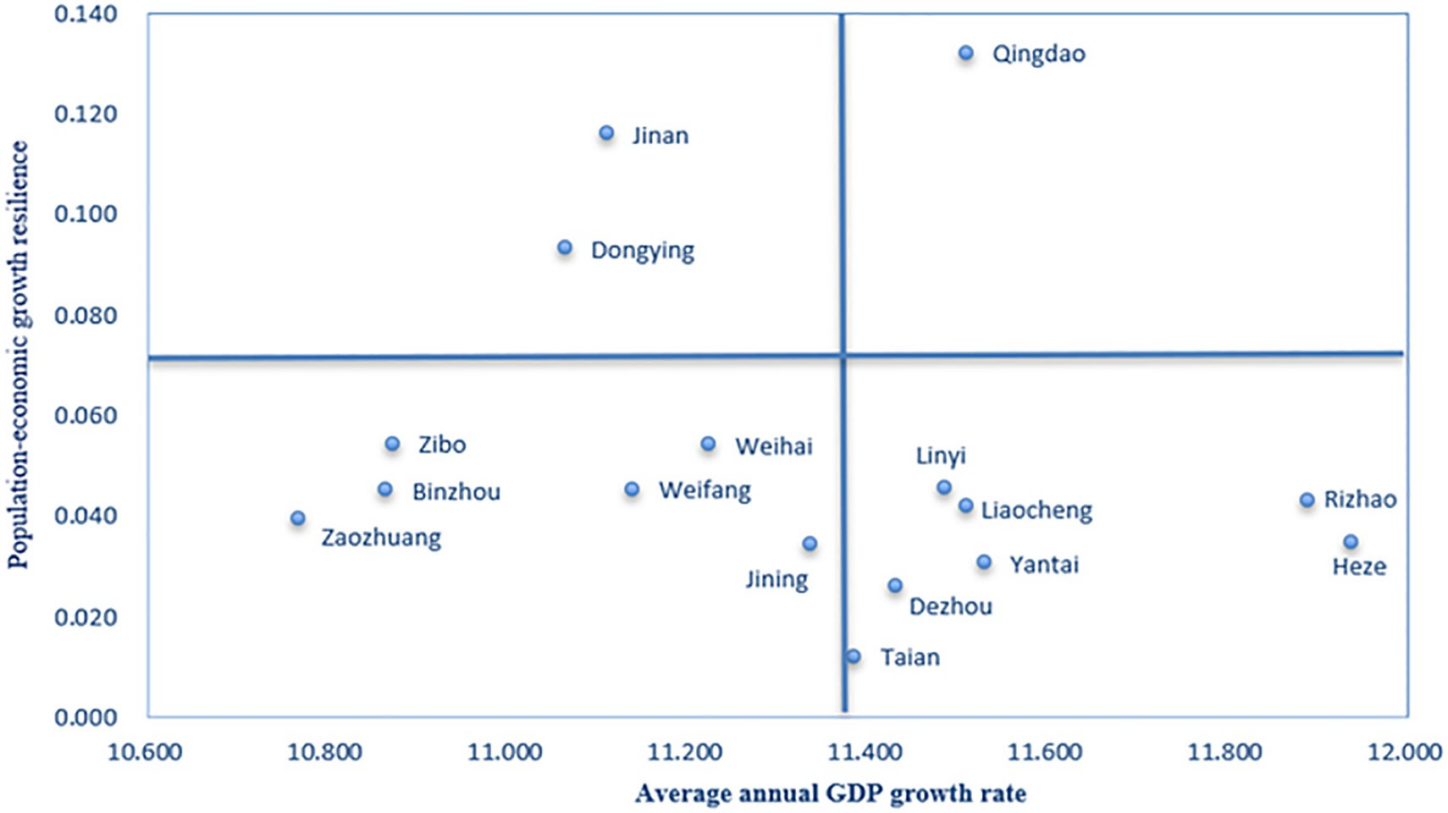
The elasticity of population-GED for 16 cities in Shandong province.

Fig 5 shows the elasticity of population-GED for 16 cities in Shandong province. To better reflect the characteristics of the CD_PGE in the province’s 16 prefectures and cities, the elasticity of population and GED in the 16 prefectures and cities was divided into four types of regions using a natural fracture method. The first category is regions with high green economic growth rates and high elasticity of population green economic growth. The region has the highest degree of coordination between population and green economy, with green economic growth being the strongest driver of population expansion, but this expansion requires sustained GED through technological innovation and improved labor productivity. Qingdao is the only city in Shandong Province in this category. The second category consists of regions with strong green economic growth rates and low green economic growth resilience of their populations. These regions have low coordination between population and green economic development, with strong GED but relatively modest population growth. The main reason is that such cities lack location advantages and are subject to resource constraints or siphon-off effects that make population outflow trends apparent. Among the Shandong cities in such a zone are Tai’an, Dezhou, Yantai, Linyi, Liaocheng, Rizhao and Heze. The third category is regions with low green economic growth rates and low elasticity of population green economic growth. Green economic growth in these regions has a weak effect on the population, so it is necessary to formulate talent development strategies to attract talent and boost GED. The cities in Shandong are Jining, Weihai, Weifang, Zibo, Binzhou and Zaozhuang. The fourth region is the one with low green economic growth rate and extreme elasticity of green economic growth of the population. Such areas have a higher degree of CD_PGE, and GED is a strong driver of population, so it is necessary to attract talent by prioritizing GED to drive population growth. Jinan and Dongying are the cities in this region in Shandong province.

### Analysis of the spatial evolution characteristics of population and GED in terms of geographical concentration

#### The demographic and GED agglomeration patterns of the three economic circles have remained mostly unchanged

**Fig 6:**
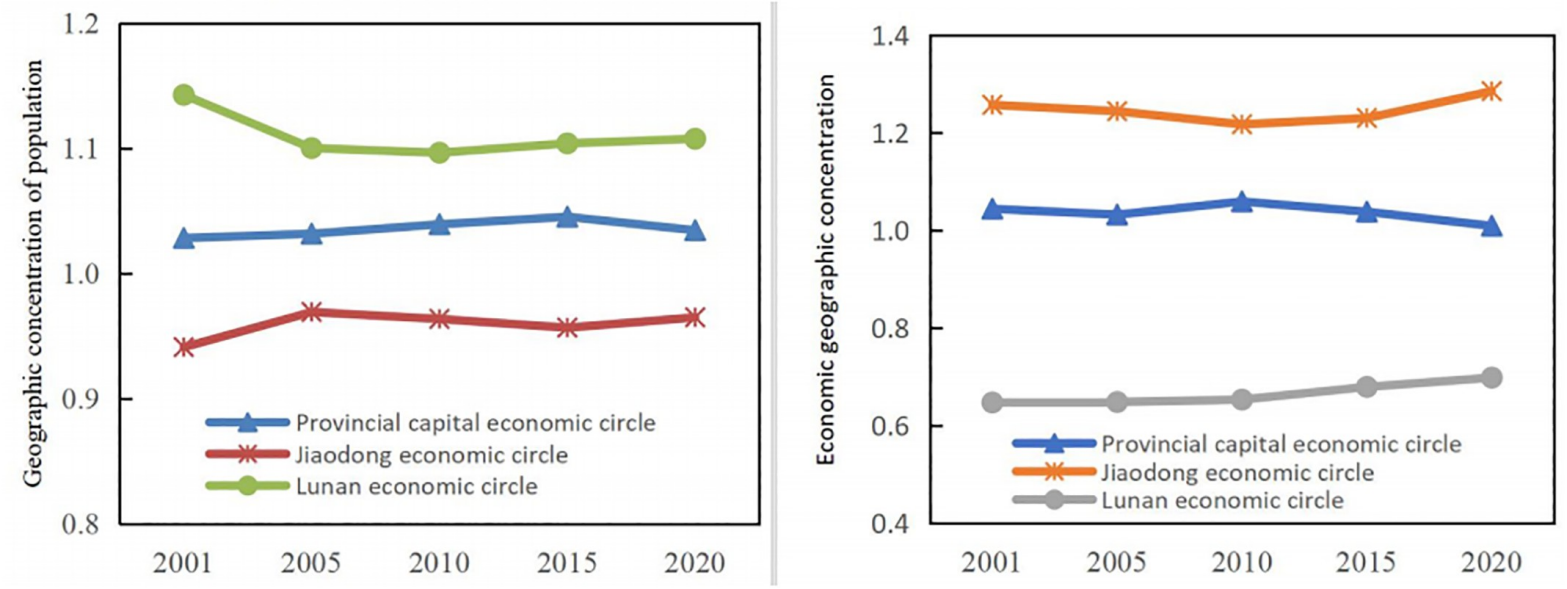
Geographical concentration of population and GED in the three economic zones of Shandong province from 2011 to 2020.

Fig 6 shows the geographical concentration of population and GED in the three major economic circles of Shandong province during 2011–2020. From the perspective of geographical concentration of population, the southern Shandong economic Belt, the provincial capital Economic Belt and the Jiaodong Economic Belt have all been characterized by a downward trend from 2001 to 2020, and the geographic concentration of population in the three economic zones has remained relatively stable over the past two decades. Spatial differentiation in the geographical concentration of Shandong’s green economy is evident, with strong GED patterns in the east and weak ones in the west standing out. From 2001 to 2020, the geographic concentration of Shandong’s GED showed a downward trend in the Jiaodong economic circle, the provincial economic circle and the southern economic circle. Within each economic band, there is a certain upward trend in the geographic concentration of the GED over time, but the magnitude of the increase is small.

#### Significant differences in geographic concentration of population and GED between cities

Table 3 reports the geographic concentration of population and GED in the municipalities of Shandong province from 2001 to 2020. Overall, most cities and towns have seen a gradual increase in population and green economic geographical concentration over time, but some cities have seen a downward trend, such as Yantai, Dezhou and Tai’an.

**Table 3:** Population and green economic geographic concentration of 16 cities from 2001 to 2020.

Cities	Geographic concentration of population					Green economic geographic concentration			
	2001	2005	2010	2015	2020	2001	2005	2015	2020
**Jinan**	1.21	1.25	1.28	1.34	1.41	1.86	1.68	1.66	2.15
**Qingdao**	1.19	1.28	1.31	1.30	1.39	1.96	2.03	2.08	2.38
**Zibo**	1.22	1.26	1.25	1.26	1.23	1.93	1.97	1.75	1.33
**Zaozhuang**	1.36	1.34	1.35	1.37	1.32	0.99	1.14	1.12	0.82
**Dongying**	0.39	0.42	0.42	0.41	0.41	1.03	1.21	1.06	0.78
**Yantai**	0.83	0.86	0.84	0.82	0.80	1.16	1.20	1.17	1.22
**Weifang**	0.93	0.93	0.93	0.93	0.91	0.82	0.76	0.81	0.79
**Jining**	1.27	1.19	1.17	1.18	1.16	0.99	0.93	0.89	0.87
**Taian**	1.20	1.18	1.17	1.16	1.10	0.94	0.91	1.03	0.77
**Weihai**	0.81	0.87	0.80	0.78	0.78	1.88	1.77	1.31	1.13
**Rizhao**	0.89	0.86	0.86	0.87	0.86	0.72	0.66	0.79	0.81
**Linyi**	1.01	0.96	0.96	0.97	1.00	0.59	0.58	0.55	0.61
**Dezhou**	0.89	0.88	0.89	0.89	0.84	0.64	0.66	0.67	0.64
**Liaocheng**	1.09	1.07	1.10	1.07	1.08	0.60	0.65	0.75	0.58
**Binzhou**	0.66	0.65	0.64	0.64	0.63	0.53	0.58	0.61	0.56
**Heze**	1.14	1.13	1.12	1.12	1.13	0.30	0.30	0.49	0.62

The spatial distribution of the geographic concentration of people in cities in Shandong Province has remained relatively stable over the past two decades. The population of the province is mainly concentrated in the central and southern regions, showing a characteristic central-periphery spatial distribution, with Qingdao and Jinan as the centers. The first-tier cities in 2020 are Jinan, Qingdao, Zibo, Zaozhuang and Jining, which have significantly higher population concentrations than other cities. Second-tier cities include Liaocheng, Tai’an, Heze and Linyi. The third-tier cities are Dezhou, Weifang, Yantai, Weihai and Rizhao. Dongying and Binzhou are in the fourth echelon and have significantly lower geographic concentrations of people than other cities. Among them, Jinan Island, which has the highest geographic concentration, has 3.4 times the population of Dongying.

The geographic concentration of economies in most cities is gradually increasing, but so is the gap between cities. The two central cities with the highest green economic geographic concentration in 2020 are Jinan and Qingdao. Yantai, Weihai and Zibo have the lowest green economic geographical concentration, and 11 other cities have the lowest green economic geographical concentration. Among the 16 cities, Qingdao has the highest green economic geographical concentration, 4.2 times that of Binzhou.

### Analysis of the spatial evolution properties of population and GED in terms of the degree of coupling coordination

Population geographical concentration and green economic geographical concentration can only reflect the spatial distribution and agglomeration of population and GED. The coupling index of population and GED effectively combines the population development index and the GED index, and can further reflect the mutual relationship between population development and GED in space, as well as its internal effects and evolutionary processes.

According to Eq 2, the coupling index of population and GED of 16 cities in Shandong Province from 2001 to 2020 was calculated and classified into five types by referring to related studies: Population polarization type (> 2), population advance type (1.5,2), population and green economic coordinated development type [0.67,1.5], green economic advance type [0.5,0.67) and green economic polarization type (<0.5).

#### The coupling between population and green economy is growing

The trend of population development and progress is stronger in most cities than the rate and trend of green economic development, indicating that cities belonging to polarised areas of population development have evolved into areas of population progress. For example, Jinan, Zibo and Weihai have evolved from areas of advanced GED to areas of coordinated population and GED; Dongying has evolved from a polarized GED zone to an advanced GED zone, while Weihai has gradually evolved from a polarized GED zone to a coordinated development zone in terms of population and GED. The rate and trend of green economic clustering in a small number of cities is stronger than that of population clustering. For example, Heze City, which was a population-polarized area until 2015, will evolve into a population-advancing area by 2020.

#### The regional scope of the coupling and coordination of the spatial distribution of population and green economy is gradually expanding

In 2001, half of the province’s cities were in a state of coordinated demographic and GED; The number of areas with coordinated population and GED increased to 10 in 2010, 11 in 2015 and nine in 2020. Among them, the type of coordinated development in Qingdao and Linyi has not changed over the past two decades; As green economic agglomeration is faster than population agglomeration, Yantai has evolved from a population and green economy developing in concert to an advanced green economic region. Liaocheng and Zaozhuang, on the other hand, have evolved from a population and green economy coordinated development type to a population advanced region as population development has outpaced GED.

#### The CD_PGE is relatively concentrated

From the functional area perspective, the CD_PGE is mainly concentrated in the economic circles of provincial capital cities. In 2020, five of the seven cities in the economic circle of the provincial capital are in a state of coordinated population and GED, with relatively high levels of coordinated population and GED. Under the guidance of the provincial economic circle strategy, Jinan’s driving force and factor agglomeration have been continuously enhanced, and the level of integrated development has been continuously improved, which has contributed to the balanced development of the internal population and green economic agglomeration. Three of the five cities in the Jiaodong economic circle are in the process of coordinated population and green economic development, namely Weihai, Weifang and Rizhao. The other two cities (Qingdao and Yantai) are in the green economic advanced region. Qingdao and Yantai, the first and third cities in the province, have a high level of GED but a relatively low level of population development due to their superior geographical location. Of the four cities in the Lunan economic circle, only Jining is in the process of coordinated population and GED, while the other three cities are in the process of population advance. The GED base of the South Shandong Economic Circle is relatively weak, with no seaport and a lack of a core city of absolute strength. Under the influence of these unfavorable factors, the level of CD_PGE is relatively low, and the green economic agglomeration of most cities is lower than the population.

#### CD_PGE with distinct spatial levels

The harmonious development index for population and green economy is divided into 4 types using the natural break-point method. The spatial distribution of the Co-ordinated Development Index of cities in Shandong Province is characterized by high values in the eastern and central regions and low values in the northern and southern regions, with a distinct spatial hierarchy. In 2001, the high value area (1.34,1.54) was mainly distributed in Jinan, Qingdao and Zibo. The median area (0.82,1.30) is mainly distributed in east Shandong and South Shandong, which are represented by 6 cities, Yantai, Weihai, Weifang, Tai ’an, Jining and Zaozhuang, respectively. The remaining area is a low value area (0,0.82). By 2020, the high-value area (1.51,1.83) is still concentrated in Jinan and Qingdao, the median area (0.88,1.51) is mainly distributed in Zibo, Yantai, Weihai, Tai ’an, Jining and Zaozhuang, and the rest of the cities are low-value areas (0,0.88). In 2020, Qingdao ranked first in the province with 1.82, while Jinan and Zibo ranked second and third with 1.74 and 1.28, respectively. Its cities have relatively low values, especially Dongying and Binzhou, which have the lowest values at 0.57 and 0.59, respectively. The maximum in Qingdao and the minimum in Dongying reach 1.25, a difference of 3.2 times, and the difference is significant.

In short, the two core cities of Qingdao and Jinan maintain a leading position in the coordinated development of spatial agglomeration. In recent years, with the progressive development of society, most cities in the province are in an advanced process, and with significant progress in population and GED, the degree of differentiation between cities has gradually decreased and the advantages of balanced regional development have become prominent.

In summary, the spatial characteristics of CD_PGE in Shandong Province are as follows. Population development levels generally show a high distribution trend in the east, central and southwest, and a low distribution trend in the north. The surrounding areas, centered on Jinan and Qingdao, have relatively high levels of GED, while those in the southeast and southwest have relatively low levels. The degree of polarization in population and GED is gradually decreasing, and the trend of balanced development is increasing. The index of the CD_PGE is gradually increasing, and the spatial distribution is characterized by high values in eastern and central regions and low values in northern and southern regions.

### Analysis of the evolution of spatio-temporal dynamic patterns in the CD_PGE in Shandong Province

#### The spatially agglomerative character of CD_PGE is evident

Table 4 reports the results of the local autocorrelation test for the index of CD_PGE. The global spatial Moran’s I value for the CD_PGE in Shandong Province from 2001 to 2020 are all positive and pass the significance test at 1 percent. Population development and GED showed significant positive spatial correlation, and that the degree of coupling coordination has a distinct spatial agglomerative character in the region. In terms of time, the global Moran’s I value, which measures the degree of CD_PGE in Shandong Province, has shown a clear trend of fluctuation and rise over the past two decades, rising from 0.032 in 2001 to 0.104 in 2020, indicating that the regions with high and low population and green economic coordination in Shandong Province present spatial agglomeration characteristics. Moreover, the agglomerative properties gradually increase with time.

**Table 4:** Moran’s I and test results for the degree of global spatial coordination of population and GED couplings.

Year	Moran’s I	Z-Value	P-Value
2001	0.032	2.854	0.004
2002	0.028	2.795	0.005
2003	0.026	2.769	0.006
2004	0.025	2.714	0.007
2005	0.024	2.723	0.006
2006	0.027	2.813	0.005
2007	0.032	2.841	0.004
2008	0.029	2.777	0.005
2009	0.033	2.764	0.006
2010	0.042	2.783	0.005
2011	0.051	2.881	0.004
2012	0.056	2.969	0.003
2013	0.061	3.051	0.002
2014	0.059	2.983	0.003
2015	0.067	3.144	0.002
2016	0.062	3.022	0.003
2017	0.064	3.042	0.002
2018	0.064	3.024	0.002
2019	0.107	3.807	0.000
2020	0.104	3.74	0.000

#### The population and GED of most cities show positive agglomeration characteristics

**Fig 7:**
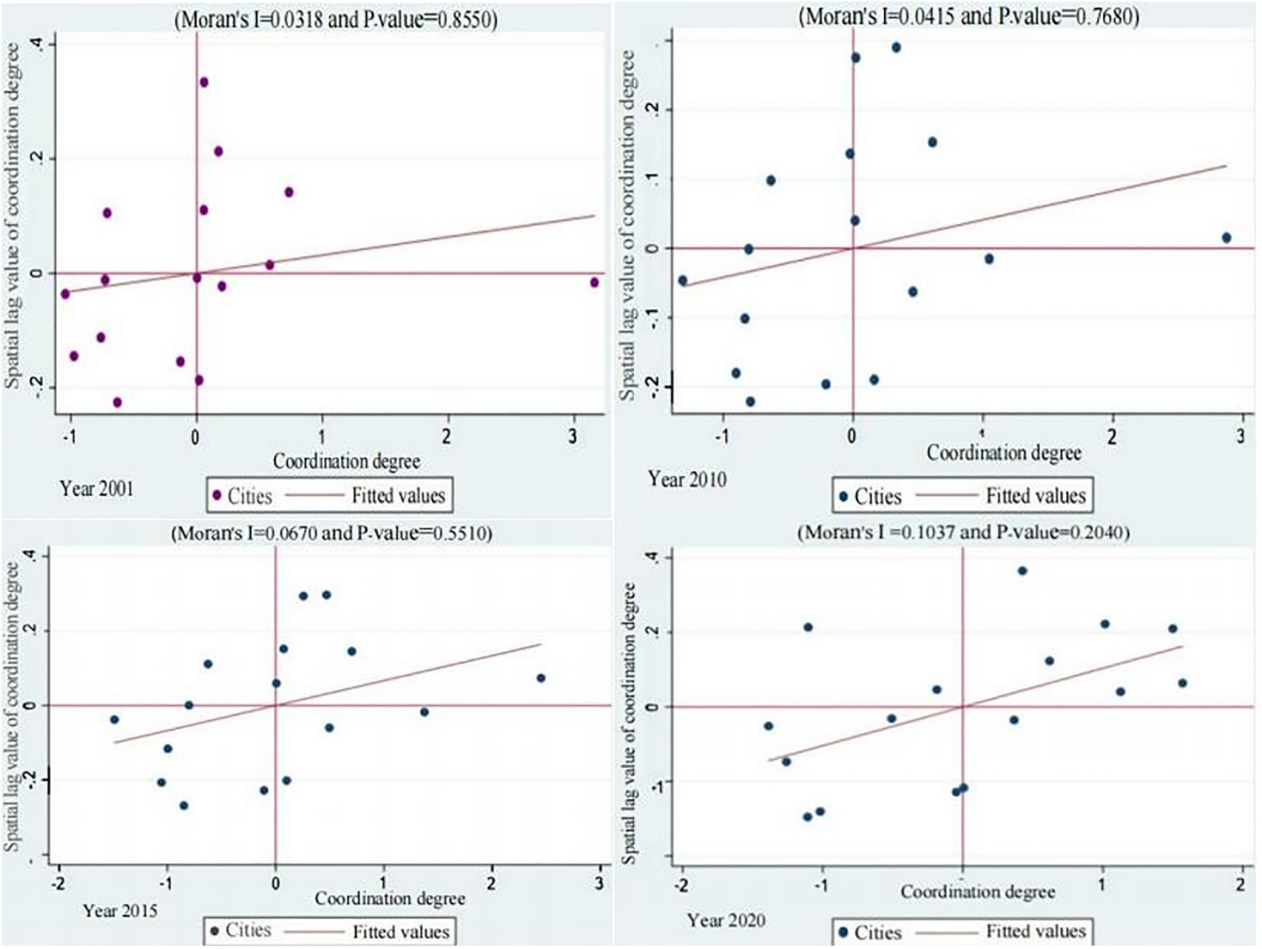
The Moran scatter plots of the coupling of population and GED in Shandong Province from 2001 to 2020.

Fig 7 reports Moran scatter plots of the CD_PGE in Shandong Province from 2001 to 2020. The figure is divided into four quadrants centered on the corresponding means, corresponding to HH, LH, LL, and HL for the inter-city coupling. Most of the points in the scatter plot are distributed in the first and third quadrants, indicating that the degree of CD_PGE between cities in Shandong Province is mostly a positive agglomeration type of HH or LL, which demonstrates the positive agglomeration characteristics of cities.

#### The degree of CD_PGE between cities has a clear spatial dependence

Table 5 reports the spatial agglomeration-type distribution of the CD_PGE in Shandong Province from 2001 to 2020. It can be seen that in 2009, HH type cities accounted for the highest proportion, 40.74 percent, followed by LL type cities, 35.19 percent, LH type cities, and HL type cities, 24.07 percent. In 2019, the percentage of HH and LL cities was 36.11 percent and 38.89 percent, respectively, while the total percentage of LH and HL cities was 25 percent. Over the past two decades, the spatial layout of urban types is relatively stable, with a significant increase in the fraction of LL and HH types and a decrease in the fraction of HL types. The non-uniform distribution of coordination degree further indicates that CD_PGE has strong spatial dependence and clustering occurs in geographically close cities. Cities with high level of CD_PGE are spatially closer, while cities with low level of CD_PGE are spatially closer.

**Table 5:** The spatial agglomeration-type distribution of CD_PGE in Shandong Province.

Type	2001	2010	2015	2020
**HH**	5	5	6	6
**LH**	1	2	2	2
**LL**	6	6	5	6
**HL**	4	3	3	2

It can be seen that HH and HL type cities are mainly concentrated in the central and northern parts of Shandong, while LH and LL type cities are mainly concentrated in the eastern and southern parts of Shandong. In the last two decades, the coupling of population and green economic growth in Shandong Province has shown clear features of spatial agglomeration evolution. The HH type cities show a slight increase, the LL type cities are basically stable, and the HL and LH type cities show a decentralized evolution over time, mainly around the LL type cities.

#### The CD_PGE between cities is clearly characterized by the evolution of the time series

**Fig 8:**
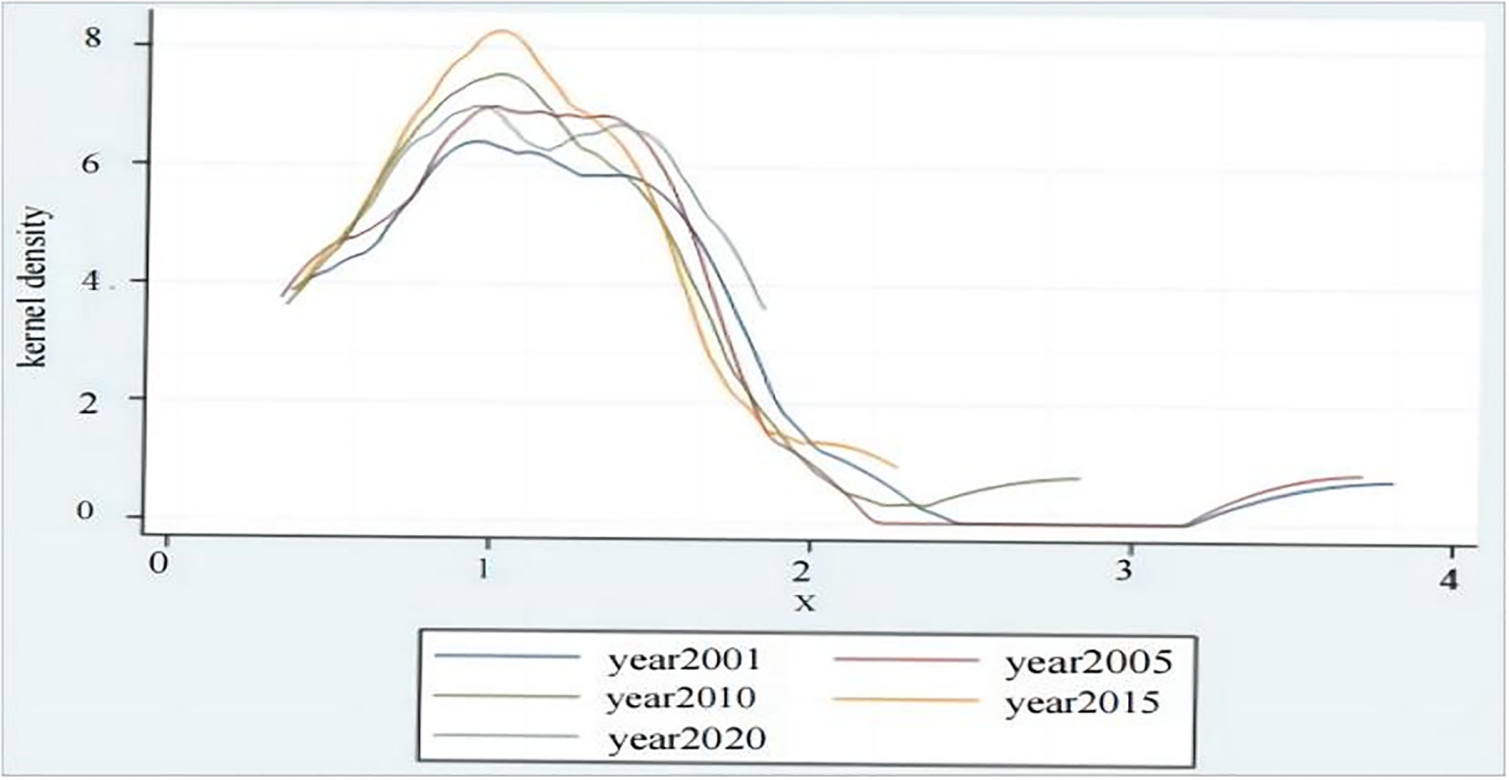
The kernel density curves for the degree of CD_PGE for the 16 cities.

Fig 8 shows the kernel density curves for the degree of CD_PGE for the 16 cities. The overall shape of the curve changed from single peak to slight multi-peak, showing that the gap of CD_PGE degree in 16 cities had a trend of gradually widening, and the trend of polarization evolution of the degree of coupling between cities has increased. Curve kurtosis tends to flatten out and height decreases slowly, showing that the extreme values of the coupling of the province’s population and green economic growth are becoming less coordinated. The tails on both sides of the curve have a tendency to gradually shrink inward, especially the contraction on the right side is more obvious, showing that the high value region and the low value region of the coupling coordination degree have a large volatility, but the volatility of the high value region is shrinking. There is little change in the position of the curve, which proves that the coordination degree between population and GED in Shandong Province is relatively stable, in agreement with previous analyses.

From the above analysis, it is concluded that GED is positively correlated with population clustering, that is, the higher the level of GED, the stronger the effect of population clustering, and the lower the level of GED, the higher the loss of population. Qingdao, for example, relies on its favorable geographical location, dense transportation lines, continuous optimization of its industrial structure, continuous GED and continuous population clustering. Jinan, as the provincial capital, relies on the advantages of Mi-zi transportation, and its GED rapidly drives the overall development of the surrounding area, attracting a large number of businesses and industries to settle there. At the same time, the continuous introduction of outstanding talent and corresponding support policies, as well as the continuous accumulation of population, can promote the development of industries and generate more innovative forces and development momentum. It will also play a driving role in the GED and social progress of the provincial capital city’s economic circle, forming a trend of mutual green economic and demographic development. As a place with good GED, Weihai and Weifang rely on the rapid transformation and upgrading of industry, GED development momentum is rapid, forming investment highlands, gathering talents and funds, attracting population by industry, and driving green economic progress by population. With slow GED, Zaozhuang mainly develops resource-intensive industries such as coal resources. However, GED has slowed in recent years due to resource exhaustion, environmental protection and industrial transformation, and the overall regional employment situation is grim. The number of migrant workers increased, the population continued to move out, the power of consumption weakened, and the degree of green economic clustering gradually decreased. It can be seen that GED and population clustering interact with each other, indicating a synergistic development relationship.

## Analysis of factors affecting CD_PGE

### Data inspection and model selection

#### Sequence stationarity test

Table 6 reports the results of the unit root test using the IPS test method. The panel data contains both temporal and cross-sectional dimensions, so it is necessary to check whether there is a root of unity in the sequence to ensure stationarity of the regression data and thus avoid spurious regressions. The test results show that the sequence of first-order differences of the selected variables is stable up to a significance level of 5 percent. Therefore, it is necessary to further explore whether there is a long-term equilibrium between the variables, that is, whether the residuals of the equation regression are stable, in order to ensure the accuracy of the equation regression results and thus to perform a co-integration test on the panel data. The residual-based DF and ADF tests, both of which have p-values of 0, reject the null hypothesis that each series of regression residuals has a root of unity, showing that there is a cointegration relation between the first-order single-integral variables and that the original equations can be estimated directly.

**Table 6:** The unit root test results.

variables	Observations	Individual intercept		Individual intercept and trend	
		Original value	First difference	Original value	First difference
**CI**	320	1.000	0.000	0.000	0.000
**CAPI**	320	0.000	0.001	0.000	0.000
**HUMAN**	319	0.000	0.000	0.000	0.000
**GOV**	320	1.000	0.000	0.000	0.000
**ROAD**	320	0.000	0.000	0.000	0.000
**IS1**	320	1.000	0.000	0.500	0.000
**IS2**	320	0.000	0.000	0.000	0.000

#### Measurement model test

Panel data model estimation methods include mixture regression models, fixed effect models, and random effect models, so it is necessary to choose the estimation method, and the choice of the model requires the following three steps. First, comparing the fixed-effect model to the mixture model, the F-test significantly rejects the null hypothesis, indicating that the fixed-effect model outperforms the mixture model. Second, comparing the random effects model to the mixture model, the BP-LM test significantly rejects the null hypothesis, indicating that the random effects model outperforms the mixture model. Finally, comparing the fixed effect model with the random effect model, the results of the Hausman test show that the fixed effect model should be used.

### Empirical results and analysis

Table 7 reports the results of regression estimation for the panel data. According to the regression results of the random effect and fixed effect models, after controlling the influence of GED level and industrial structure on the CD_PGE, the four influencing factors variables of human capital level and material capital level, government intervention intensity and transportation infrastructure all passed the significance level test. In particular, the physical capital level variable passes the test at a significance level of 1 percent and the sign of the coefficient is negative, indicating that fixed-asset investment per capita is negatively correlated with the population green economic coupling index. The increase in fixed-asset investment per capita will clearly inhibit the CD_PGE, and will exacerbate the inconsistencies in population development and GED. All the human capital variables pass the significance test at the 1% level, and the sign of their coefficients is negative, indicating that an increase in the level of human capital in the area is not conducive to the coordination between the population and the green economy of the city. From the point of view of the magnitude of the regression coefficient, the regression coefficient for the level of human capital is higher than that for physical capital, which has a larger impact on the index of CD_PGE. And changes in the level of human capital have a relatively greater impact on the CD_PGE. Therefore, as an alternative indicator of market mechanism, the empirical results of physical capital and human capital are consistent with the research hypothesis, indicating that in the process of guiding factor flow and resource allocation, the role of market mechanism will exacerbate the inconsistency in the spatial distribution of population and green economy in Shandong Province, which is not conducive to the CD_PGE between regions.

**Table 7:** Panel model regression results.

Variables	RE	FE
	DI	DI
**HUMAN**	-0.0501***	-0.0635***
	(-4.8316)	(-6.6309)
**CAPI**	-0.0282**	-0.0430***
	(-2.1212)	(-3.5521)
**GOV**	-1.6649***	-1.4025***
	(-6.4170)	(-5.8747)
**ROAD**	0.0643*	0.0374*
	(1.8612)	(1.1952)
**PGDP**	-0.0193**	-0.0750***
	(-2.0305)	(-3.1795)
**IS1**	0.0095***	0.0088***
	(7.3874)	(7.5389)
**IS2**	0.0117***	0.0099***
	(7.2404)	(6.7863)
**_cons**	0.4605***	0.5423***
	(5.0093)	(6.9610)
**N**	319	319
**adj. R2**		0.332

t statistics in parentheses

* p < 0.1,

** p < 0.05,

*** p < 0.01

The level of transportation infrastructure passed the significance test with a significance level of 10 percent, and its coefficient was also positive, indicating that the improvement of transportation infrastructure level is positively correlated with the CD_PGE of cities. The development of transportation facilities will increase the coupling index of population and green economy, indicating that the improvement of transportation infrastructure in Shandong Province is more conducive to accelerating the flow of demographic factors, alleviating the inconsistency of spatial distribution between population and green economy in Shandong Province to a certain extent, and thus promoting the CD_PGE. The variable of the intensity of fiscal intervention also passes the significance test with a negative sign, indicating that government intervention and policy tendencies will accelerate population and green economic clustering in policy regions. However, due to the different convergence rates of the two, the coordination between population and green economy will be reduced, which is consistent with the theoretical expectation assumption.

When the market mechanism plays its role, it will direct the flow of resources to economically developed regions. As a factor in the workforce, the population will move to places where the green economy is clustered, provided there are no institutional barriers. However, the population agglomeration will indirectly lag behind the green economic agglomeration due to the lag in population mobility, which will increase the spatial incongruity in the distribution of population and green economy, which is not conducive to the CD_PGE. The government is committed to improving infrastructure for regional connectivity, which is conducive to population migration and mobility, and will go some way to alleviate inconsistencies in the distribution of population and green economy in Shandong, so that regional populations and economies will tend to harmonize. During the phase of rapid industrialization, some of the key regional priority development policy factors adopted by Shandong will aggravate the inconsistent spatial patterns of urban population and green economy. In the course of GED, especially during the period of rapid industrialization, while promoting GED, it also promoted the concentration of population in the region. However, government policies are more conducive to rapid green economic agglomeration. Moreover, the hysteresis of population agglomeration further widens the spatial inconsistency between population and green economy, thus reducing the level of CD_PGE.

Table 8 reports the results of the robustness test. Empirical results show that the regression coefficients for material and human capital accumulation are significantly negative in each segment, indicating that market factors adversely affect CD_PGE. The regression coefficient for government intervention is significantly negative in each segment, indicating that excessive government intervention is not beneficial for CD_PGE. The regression coefficients for transportation infrastructure are significantly positive in each sub-segment, indicating that the development of infrastructure promotes the improvement of CD_PGE levels. The results of panel quantile regression generally agree with those of fixed- and random-effects panel models, validating the robustness of the models constructed in this study and the empirical results. In addition, it can be seen from the regression results of different loci that the regression coefficients of physical capital level and transportation infrastructure level gradually increase significantly with the increase of loci, indicating that the influence of human capital level and transportation infrastructure level on cities with high coupling degree of population green economy is much greater than that of cities with low coupling degree. The regression coefficients for the level of human capital and the level of government intervention fluctuate with the number of sub-points and the range of variation is relatively small, indicating that the level of human capital and the level of government intervention have some influence on the level of coupling between cities with different levels of coupling. The regression coefficients for the level of government intervention and transportation infrastructure are significantly higher than those for human and material capital, indicating that the contribution of government intervention to the level of coordinated development of Shandong’s population and green economy is gradually increasing.

**Table 8:** The robustness test results.

Variables	q25	q50	q75
**HUMAN**	-0.0902***	-0.0829***	-0.1095***
	(-6.7536)	(-3.5174)	(-3.0806)
**CAPI**	-0.1453***	-0.2664***	-0.3218***
	(-4.4700)	(-4.2075)	(-5.9652)
**GOV**	-2.1872***	-1.5871***	-2.4322***
	(-4.6517)	(-2.8463)	(-3.2602)
**ROAD**	0.7756***	1.0726***	1.3020***
	(7.2911)	(7.3204)	(10.9856)
**PGDP**	-0.1137***	-0.1773***	-0.2964***
	(-4.4700)	(-3.1028)	(-4.8236)
**IS1**	0.0100**	0.0259***	0.0290***
	(1.9701)	(2.8534)	(4.4565)
**IS2**	0.0148***	0.0255***	0.0251***
	(2.9751)	(2.6861)	(3.3644)
**_cons**	0.2895	0.3839	0.6830*
	(1.4435)	(1.1240)	(1.8882)

t statistics in parentheses,

* p < 0.1,

** p < 0.05,

*** p < 0.01

## Discussion

The relationship between population distribution and regional GED has been the focus of cross-sectional research in the fields of demography, geography and economics. Endogenous growth theory argues that there is an interwoven endogenous relationship between population and GED. As an important driver of GED, the redistribution of population between regions will have a profound impact on the speed and pattern of regional GED. Meanwhile, the inter-regional GED gap will also lead to a spontaneous realignment of population distribution. In the macroeconomic context of imbalanced inter-regional economic development, the prominent urban-rural duality problem in China, and the huge mobile population, it is of great theoretical and practical importance to explore the relationship between population distribution and GED and its influencing factors in the case of Shandong Province.

The interaction between population development and GED in Shandong is strong, but at the same time, there are some problems, such as the uncoordinated development of the two systems and large regional development differences. It is important to address the bottleneck of incongruity between regional population and green economic development at various points and in various aspects, and jointly create a sound situation for the coordinated development of strong and active regional population and green economic quality, and then realize a new pattern of regional development. Therefore, the following proposal is made in this paper.

### Overall coordination and optimization of population distribution and GED pattern

One is to optimize the green economic spatial layout and raise the level of GED in northwest and southwest Shandong. It is necessary to further expand investment in northwest and southwest Shandong, especially in Liaocheng, Dezhou, Heze and other places with relatively weak GED, and promote the development of regional economies and population clusters through industrial support and industrial transfer. Second, we should focus on population transition and innovative development ideas, Xi said, adding that the eastern region will bring in high-quality talent to promote the adaptation and development of the region’s population and green economy, and maximize the combined force of population and green economy. Shandong’s central and southern regions should improve the quality of their populations, strengthen the training of people’s skills, broaden employment channels, accelerate the process of urbanization and realize the transfer of surplus rural people. Third, a coordinated development mechanism should be established at the provincial level with a focus on regional development. The decision-making level should take into account the huge differences in the development of each city from the actual situation and make full use of the advantages of policy to play a coordinated development role. Fourth, we will strengthen the driving role of the two-core structure, with Jinan and Qingdao as the core, and build a new model of coordinated development. Fifth, the network of urban systems should be strengthened to enhance the attractiveness of small and medium-sized cities. Combined with this research, it can strengthen the overall development level of provincial cities and highlight the engine role of core cities in urban systems. We will develop the resource endowments of different cities and highlight the development of multiple cores and different fields.

### Optimize the distribution of factors and enhance the promoting role of factors in the CD_PGE

First, invest more in education and focus on personnel training. This paper finds that improving human capital levels plays an important role in promoting regional economic development. Shandong has 153 colleges and universities, but most of them are concentrated in Jinan and Qingdao, especially in some relatively backward areas with poor educational resources, which has severely affected regional economic development. Therefore, we should strengthen investment in education in relatively backward regions, guide cooperation between higher education resources and less developed regions, improve the overall quality of the population, narrow the regional gap by relying on innovative resources, and achieve coordinated development of regional populations and green economies. In addition, personnel training programs are implemented according to local conditions to provide a platform for talent development. We will continue to promote innovation in personnel policies, concepts, forms and services, and let the market play a decisive role in the allocation of human resources. Improving the recruitment, attraction, hiring and retention of talent through clustering effects will give full play to the effectiveness of programs that attract talent and those that bring it in, promote industrial development, activate economic vitality and promote the rapid development of the local economy.

Second, improve infrastructure, optimize industrial structure and raise the level of comprehensive regional development. Shandong Province has a wide range of regional resource endowments, population and GED levels, and the improvement of infrastructure and the optimization of industrial structure have clearly contributed to the improvement of regional GED levels. Provincial governments should increase investment in urban infrastructure, optimize the environment for GED, strengthen the support of economically leading cities to relatively backward economies, and raise the level of comprehensive regional development.

Third, strengthen regional ties and improve transfer payment capacity. We will give priority to areas with weak economic development, such as finance, land and human resources, and strengthen our capacity to transfer payments. We will strengthen regional links, build provincial transport network systems and reduce the time cost of economic and trade interactions. Arrange people from economically developed areas to guide urban development in economically backward areas and encourage them to invest locally.

Fourth, use our strengths to accelerate industrial transformation and upgrading. Learn from the advanced development experiences of developed regions while accelerating the transformation and upgrading of primary and secondary industries, taking into account local resource endowments, development foundations and distinctive industrial advantages. Improve industrial distribution, strengthen and improve manufacturing, and foster, develop and expand tertiary industries.

### Optimize institutions and mechanisms, and strengthen the government’s regulatory role in the coordinated development of population and regions

The first is to adapt to local conditions and promote balanced population and GED. Economic development can react to the distribution of population, and economically developed areas have a strong attraction for the population. But in some areas, the economy is developing more slowly and the population is growing. We can increase preferential policies to attract enterprises to settle here, improve market institutions and mechanisms, and promote the development of private enterprises and micro, small and medium-sized enterprises. Provide a large number of jobs and form an effective workforce. At the same time, we will increase subsidies for talent and improve various security systems for talent to attract high-end talent to the country.

Second, we will promote institutional reform and promote the development of small and medium-sized cities. Combined with this study. It is necessary to promote administrative reform measures such as expanding the powers of strong counties and direct administration of counties by provinces in economically strong counties, and to formulate policies favorable to the green economic development of strong counties. We can learn from the idea of cultivating small cities in Jiangsu and Zhejiang, and expand the power of townships with sound economic foundations and large populations to promote their sustainable development. Urban integration requires differentiated policies that focus on economically strong cities, help economically weak cities and promote common development.

The third is to promote the reform of the household registration system and promote the integration of urban and rural areas. Currently, some provinces and regions have canceled agricultural and non-agricultural household registration. But rural people do not enjoy the same social security benefits as urban residents. With reference to the existing literature and in conjunction with this study, we can follow: deepen the reform of the household registration system and continue to promote the equalization of basic public services. The use of residential land as a precondition for urban hukou cannot be withdrawn from the right to manage land contracts without fully investigating and respecting the agricultural wishes of agricultural workers. We will actively promote the development of rural education, health care and social security, and close the gap between urban and rural areas.

Fourth, we will build a regional common market and establish an orderly market-based green economic system. The pursuit of higher-quality regional integration requires restructuring the fragmented market to create a unified market with orderly competition. Remove barriers that are incompatible with the development of a single market, reduce restrictions that are detrimental to the free movement of factors, and eliminate unnecessary regulatory practices that impede fair competition. In building a unified market, the government should play an overall coordinating role, provide a level playing field for cooperation and competition, and let resources flow freely to the maximum extent. The construction of a unified market will not be achieved overnight, and the promotion of market integration cannot be divorced from the characteristics of realistic development. It is necessary to promote the process of market integration by regions and stages, find a breakthrough in each economic circle as a starting point and gradually spread it throughout the province from point to point, line to line and surface to surface.

Fifth, we will improve policy supervision and interest coordination mechanisms to ensure the implementation of policies. The government should play a role in regional coordination, and policy mechanisms should be innovative and flexible. We will actively promote the construction of 5G and other information infrastructure, improve big data information platforms, and build a real-time and effective information network to provide a policy basis and an authoritative and effective coordination and communication platform for the coordinated development of the province. We will improve the mechanism for coordinating interests and establish an evaluation index system for policy implementation. Depending on the functional orientation of different regions, different evaluations and evaluations were performed. A system of rewards and punishments for policy implementation should be established to enhance the authority and efficiency of regional policy implementation.

### Adopt local policies to strengthen weaknesses and strengths to promote CD_PGE

First, for Weihai, Weifang and other population and green economic coordination areas in the Jiaodong economic circle, the industrial energy level should be expanded and the industrial spatial layout should be continuously optimized. We will give priority to ecological and green development, promote high-quality development of the manufacturing sector, develop the digital economy in an innovative way and achieve integrated development of the digital economy and the real economy. Qingdao, Yantai and other economically advanced regions need to continuously consolidate the position of traditionally favored industries, promote industrial structure optimization and upgrading, and accelerate the formation of industrial clusters. Second, we will promote high-quality economic and social development in the economic circles of provincial capital cities, and while absorbing and pooling the resources of surrounding cities, Jinan should export integrated resources to surrounding cities in an orderly manner. Jinan, Zibo, Tai’an and other areas with coordinated green economic development should further promote the interconnection of transportation infrastructure, build a modern comprehensive transportation system compatible with the urban-rural structure, industrial layout and ecological structure, and achieve coordinated population and GED. Finally, we will support medium and long-term population improvement in the southern Shandong Economic Belt region relative to the surplus population in the central cities of the economic belt. To develop the Lunan economic circle, it is necessary to promote the construction of new rural areas and urbanization, guide the transfer of surplus rural labor, promote the rapid green economic development of the region, and continuously raise the level of high-quality coordinated development of the region’s population and green economy.

In addition, the study presented in this paper still has the following shortcomings. On the basis of the objective complex relationship between GED and population development, through the identification of synchronous development and coupling coordination degree and driving force, the analysis framework of coupling and coordination development types of the two systems is constructed, and the optimization path of different types of cities is studied and judged. However, Shandong, China’s largest province in terms of population and green economy, has a distinct development process from other regions. Affected by models and evaluation criteria, it lacks a certain level of generality at the macroscopic level. Secondly, there is a certain threshold for the population system to be affected by economic and social activities. Therefore, in the future, it is necessary to pay attention to the definition of the threshold of the evaluation index so that high-quality CD_PGE can be achieved below the capacity threshold.

## Conclusions

In this paper, we study the dynamical evolution and spatio-temporal characteristics of the degree of CD_PGE in Shandong Province, China, from 2001 to 2020. We use kernel density estimation, mathematical statistical analysis, visual analysis, and spatial autocorrelation analysis in a comprehensive manner to reveal spatial heterogeneity, spatial correlation properties, and temporal evolution trends of area coordination values. Based on this, we construct a panel measurement model to analyze the factors affecting the region coordination values. The main conclusions of this paper are the following.

In terms of demographic and GED characteristics, Shandong’s demographic and GED trends are favorable, but it still faces numerous challenges. In terms of time, the aggregate population and green economy of Shandong continued to rise during the study period, and the driving effect of GED on population growth gradually increased, but the level of CD_PGE remained low. From a spatial point of view, the geographical concentration of population and green economy in Shandong Province presents distinct spatially stratified features, with strong green economic development patterns in the east and weak ones in the west. The spatial distribution of green economic geographical concentration is centered-periphery, with Qingdao and Jinan as the centers. The geographic concentration of economies in most cities is gradually increasing, but so is the gap between cities.

From the evolution of the time series and the spatial distribution of the CD_PGE, the level of the spatial distribution is clear, as the level of CD_PGE in Shandong continues to rise. During the study period, population development and progress trends were stronger than GED rates and trends in most cities in Shandong. The regional extent of the CD_PGE is gradually expanding, and the areas of coupling development are relatively concentrated. From the functional area perspective, the CD_PGE is mainly concentrated in the economic circles of provincial capitals. The spatial distribution of the index of CD_PGE is characterized by high values in the eastern and central regions and low values in the northern and southern regions, with a sharp spatial hierarchy.

From the perspective of spatio-temporal dynamical pattern evolution of the degree of CD_PGE, the CD_PGE is characterized by significant spatial clustering, but with large regional differences. The degree of CD_PGE among cities in Shandong Province has a strong positive correlation in space, and the degree of coupling coordination has a distinct spatial agglomeration feature in the region. The degree of CD_PGE shows a tendency to gradually widen among the 16 cities, and the polarization evolution of the coupling between cities increases. The extreme values of the degree of CD_PGE are decreasing, as is the maximum gap of the imbalance between cities. Both the high and low value regions of the coupling coordination show greet fluctuations, but the high value city is relatively stable in terms of decreasing coupling coordination for the population and GED of the province.

From the point of view of influence factors, both market mechanisms and government intervention have a significant impact on the degree of CD_PGE, but the direction and extent of the influence varies. The increase in fixed-asset investment per capita and the rise in human capital, which represent market mechanisms, will clearly inhibit the CD_PGE, and aggravate the inconsistencies in the development of the population and green economy. In terms of government intervention, improving the level of transportation infrastructure is positively correlated with the CD_PGE of Shandong Province. The intensity of fiscal intervention will accelerate population and green economic clustering in policy areas, but the coordination between population and green economy will be reduced due to different clustering rates.
